# Effects of dental treatment and systemic disease on oral health-related quality of life in Korean pediatric patients

**DOI:** 10.1186/s12903-018-0552-0

**Published:** 2018-05-29

**Authors:** Ji-Soo Song, Hong-Keun Hyun, Teo Jeon Shin, Young-Jae Kim

**Affiliations:** 1 Department of Pediatric Dentistry, Seoul National University Dental Hospital, 101, Daehakno, Jongno-gu, 03080 Seoul, Republic of Korea; 2 Department of Pediatric Dentistry, Dental Research Institute, School of Dentistry, Seoul National University, Seoul National University Dental Hospital, 101, Daehakno, Jongno-gu, 03080 Seoul, Republic of Korea

**Keywords:** Child, Dental treatment, Systemic disease, Oral health-related quality of life

## Abstract

**Background:**

The findings that not only dental caries but also systemic disease can exert a negative effect on oral health-related quality of life (OHRQoL), and that dental treatment can improve OHRQoL have been confirmed in multiple studies. The purpose of this study is to investigate the impact of dental treatment on OHRQoL of Korean pediatric patients and the differences in OHRQoL between patients with and without systemic disease.

**Methods:**

All the primary caregivers of pediatric patients who underwent dental treatments under either general anesthesia or intravenous deep sedation at Seoul National University Dental Hospital completed abbreviated versions of the Child Oral Health Impact Profile (COHIP-14) and Family Impact Scale (FIS-12) surveys on OHRQOL pre- and post-treatment (average: 2.4 ± 1.7 months after dental treatment)*.* This is a case control study with patients divided into two groups according to the presence or absence of systemic disease.

**Results:**

Data from 93 pediatric patients (46 male and 47 female, average patient age: 5.0 ± 3.4 years) were analyzed to compare OHRQoL before and after treatment with the Wilcoxon signed-rank test and to calculate the effect size using Cohen’s d. All of the patients exhibited an improvement in OHRQoL (COHIP-14: *p* <  0.001, effect size = 1.0; FIS-12: *p* <  0.001, effect size = 0.7). Patients with systemic diseases demonstrated lower OHRQoL in both pre- and post-treatment surveys than patients without systemic diseases (Wilcoxon Rank-sum test, both COHIP-14 and FIS-12: *p* <  0.05). The COHIP-14 appears to have a greater impact on the FIS-12 in patients with systemic disease than those without (explanatory power of 65.3 and 44.6%, respectively).

**Conclusions:**

Based on the primary caregivers’ perceptions, dental treatment can improve the OHRQoL in Korean pediatric patients. Systemic disease results in a reduced OHRQoL, and the awareness of patients’ oral health appeared to have a greater impact on OHRQoL for family members of patients with a systemic disease.

**Trial registration:**

KCT0002473 (Clinical Research Information Service, Republic of Korea) and 22 Sep 2017, retrospectively registered.

## Background

Dental caries is the most common chronic oral disease, with a high prevalence in children and adolescents worldwide [1]. The prevalence of active caries in primary teeth was as high as 34.5%, and that of dental caries experience in primary teeth was as high as 62.2% in 2012 Korean study [2]. Oral health-related quality of life (OHRQoL) is a multidimensional concept that includes a subjective evaluation of the individual’s oral health status, functional well-being, social and emotional well-being, expectations of and satisfaction with care, and sense of self-image [3]. Its importance is widely emphasized in both research and clinical settings, given the increasing demand for active participation of patients in the treatment process, and the lack of basic treatment for certain chronic diseases (e.g., dental caries, periodontal disease) that require long-term treatment and follow-up. Nevertheless, research about the OHRQoL of pediatric patients in Korea has only recently been initiated despite the high prevalence of dental caries. The only study that has conducted a full-scale reliability and validity test in Korea was reported by Ahn et al., in which a Korean version of the Child Oral Health Impact Profile (COHIP) was used in 2236 children and adolescents aged 8−15 years [4].

The findings that dental caries can exert a negative effect on OHRQoL and that dental treatment can improve OHRQoL have been confirmed in several studies [5–9]. Pain caused by dental caries can interfere with normal masticatory function and sleep, which can inhibit normal body growth [10]. Unpleasant smiles associated with the destruction of tooth structure also can negatively influence the social life of children [11]. In addition, the perceptions and attitudes of primary caregivers on oral health influence the behavioral patterns regarding their child’s oral health [12]. Chronic disease such as dental caries in children can affect family life [13, 14]_,_ and patients with systemic disease have been shown to have low OHRQoL [15–17]. Their underlying disease may be associated with poor oral health, but they may also have difficulties maintaining their oral health and accessing adequate dental care due to underlying disease [18]. There are no studies that have been conducted to identify the relationship between dental caries and the OHRQoL of pediatric patients and their families, and to compare the OHRQoL between patients with and without systemic diseases in Korea. Therefore, this study examined the impact of dental treatment on OHRQoL of Korean pediatric patients and the differences in the OHRQoL between patient with and without systemic disease.

## Methods

### Subjects

This study involved all primary caregivers or parents of pediatric dental patients who underwent dental treatment under either general anesthesia or intravenous deep sedation at Seoul National University Dental Hospital pediatric department from February 2013 to February 2014. Five professors who were all experienced in general anesthesia and intravenous sedation in the pediatric dentistry department conducted all the dental treatments, and standardized treatment protocols were followed. Patients who received dental treatment without general anesthesia or intravenous sedation were not included in the study. The use of general anesthesia or intravenous sedation is decided by the anesthesiologist based on the physical condition of the airway and respiratory system, not on the severity of dental caries. Primary caregivers accompanying the patients on the day of treatment were invited to participate in the survey. The study was performed with the approval of the Seoul National University Dental Hospital Research Ethics Board (IRB Number: CRI12006). We fully explained the study to the primary caregivers only if they were the legal guardians of the patients and only included participants with written consent on the day of treatment.

### Study design

This study was a case control study to compare OHRQoL between the patients with and without systemic disease in each cohort. Accordingly, patients were categorized into two groups. The patient group without systemic disease did not exhibit conditions that encumbered everyday life, but required either general anesthesia or intravenous deep sedation due to dental phobia and a large number of dental caries. The group with systemic disease included patients with special health care needs, such as intellectual disability (ID), autism, or developmental disorders, as well as conditions that affect everyday life (e.g., cancer, cerebral palsy, convulsive disorders, genetic disorders, and cardiovascular disorders) [19, 20]. Cases with dental treatments that did not involve pulp treatment or restorations—including periodontal treatment, such as scaling, or minor oral surgery, such as removal of supernumerary teeth—were excluded from the study.

Primary caregivers were asked to fill out surveys on OHRQoL, pre-treatment as well as post-treatment when the patients returned for a follow-up visit. As calculated from our pilot study performed in the initial stage of this study with 20 patients (10 patients without systemic disease and 10 patients with systemic disease), the power calculation indicated that 104 cases were required to compensate for a 20% drop-out rate at 5% significance level and 80% statistical power. The pre-treatment survey included responses from 109 cases and follow-up post-treatment surveys were completed for 93 of these cases within 6 months. These cases were selected for analysis. Sixteen cases were excluded, as the patient did not have a follow-up appointment, a different primary caregiver accompanied the patient for the post-treatment visit, or the primary caregiver declined to complete the post-treatment survey. The post-treatment survey was completed in an average of 2.4 ± 1.7 months after dental treatment.

### Surveys

In order to assess OHRQoL, the Child Oral Health Impact Profile (COHIP) and Family Impact Scale (FIS) were utilized. An abbreviated version of the COHIP, “COHIP-14”, which included 10 items from the Oral Health subscale (OH) and 4 items from the Functional Limitation subscale (FL), was used in this study [21]. Similarly, the “FIS-12” scale used in the study included 5 items from the Parental/Family Activity subscale (PA), 4 items from the Parental Emotion subscale (PE), 2 items from the Family Conflict subscale (FC), and 1 item from the Financial Burden subscale (FB) [21]. Because pediatric patients requiring general anesthesia or intravenous sedation were usually younger than the target age of COHIP and FIS, these subscales and items were selectively chosen from the original questionnaires to be reasonably assessed among the primary caregivers of these patients. Several items that caregivers could not answer correctly or required an active response from the patient were removed through an active discussion between two experienced dentists [21]. The resultant subscales and items are outlined in Tables 1 and 2. The pre-treatment COHIP-14 survey assessed the frequency of issues arising from dental disorders in pediatric patients from the primary caregiver’s perspective, and the FIS-12 assessed the impact on everyday life activities and emotions of the patient and family members in the 3 months prior to the survey. For the post-treatment survey, the primary caregivers were instructed to reflect on changes post-treatment when completing both the COHIP-14 and FIS-12. Both measurements utilized a 5-point Likert scale, where the COHIP-14 and the FIS-12 ranged from 0, being “Never”, to 4, being “Almost every day”. Because the items of COHIP-14 were negatively worded, the scores in COHIP-14 were reversed [22]. The scores of the items were added to calculate subscale scores, which were then summed to obtain the finalized COHIP-14 and FIS-12 scores. The COHIP-14 score ranged from 0 to 56, while FIS-12 score ranged from 0 to 48. Higher COHIP scores and lower FIS scores corresponded to a better OHRQoL.

**Table 1:** Prevalence and mean values of the 14-item Child Oral Health Impact Profile (COHIP-14) scores before and after dental treatment (average 2.4 ± 1.7 months’ follow-up period) (*n* = 93)

	Before COHIP-14 score	After COHIP-14 score	Difference (after-before)		
	Mean(SD)	Mean(SD)	Mean(SD)	Effect size ^a^	*p-value*
COHIP-14	37.5(7.9)	45.2(7.7)	7.7(8.1)	1.0	< 0.001*
Oral Health subscale (OH)	26.0(5.4)	31.9(5.7)	5.8(6.0)	1.0	< 0.001*
Pain/tooth ache	2.7(1.0)	3.4(0.8)	0.7(1.2)	0.6	
Breathing through mouth	2.0(1.1)	2.5(1.2)	0.5(1.2)	0.4	
Discoloration of teeth	1.9(1.4)	3.4(1.1)	1.5(1.6)	0.9	
Crooked teeth or spaces	2.5(1.5)	3.5(1.0)	1.0(1.6)	0.6	
Sores or sore spots	3.4(0.8)	3.6(0.7)	0.2(0.8)	0.3	
Bad breath	2.1(1.3)	2.8(1.2)	0.6(1.2)	0.5	
Bleeding gums	3.2(1.0)	3.3(0.9)	0.1(0.9)	0.1	
Food sticking	2.1(1.0)	2.5(1.1)	0.4(1.3)	0.3	
Sensitivity to hot/cold	3.1(1.0)	3.6(0.7)	0.5(1.2)	0.4	
Dry mouth	3.1(1.1)	3.3(0.9)	0.2(1.0)	0.2	
Functional Limitations subscale (FL)	11.4(4.0)	13.3(3.0)	1.9(3.6)	0.5	< 0.001*
Trouble chewing firm foods	2.3(1.5)	2.9(1.4)	0.6(1.5)	0.4	
Difficulty eating	2.8(1.3)	3.3(1.0)	0.6(1.3)	0.5	
Trouble sleeping due to teeth/face	3.6(0.8)	3.9(0.4)	0.3(0.8)	0.4	
Difficulty keeping teeth clean	2.8(1.4)	3.2(1.2)	0.4(1.4)	0.3	

Wilcoxon signed-rank test

*Significant at α = 0.05 level

^a^Calculated using Cohen’s d (= difference / SD)

**Table 2:** Prevalence and mean values of the 12-item Family impact scale (FIS-12) scores before and after dental treatment (average 2.4 ± 1.7 months’ follow-up period) (n = 93)

	Before FIS-12 score	After FIS-12 score	Difference (after-before)		
	Mean(SD)	Mean(SD)	Mean(SD)	Effect size ^a^	*p-value*
FIS-12	15.7(9.2)	10.3(8.3)	5.4(8.3)	0.7	< 0.001*
Parental/family Activity subscale (PA)	7.4(4.8)	4.7(4.5)	2.7(4.7)	0.6	< 0.001*
Taken time off work	0.6(1.0)	0.3(0.7)	0.3(1.0)	0.3	
Required more attention	2.8(1.3)	1.9(1.5)	0.8(1.5)	0.5	
Had less time for yourself	1.5(1.4)	1.0(1.4)	0.5(1.6)	0.3	
Sleep disrupted	1.5(1.3)	0.8(1.1)	0.7(1.2)	0.6	
Family activity interrupted	1.0(1.3)	0.6(1.0)	0.4(1.3)	0.3	
Parental Emotion subscale (PE)	6.1(3.8)	4.0(3.4)	2.1(3.2)	0.7	< 0.001*
Been upset	1.3(1.2)	0.9(1.0)	0.4(1.1)	0.4	
Felt guilty	1.8(1.3)	1.2(1.1)	0.6(1.1)	0.5	
Worried about less opportunity	2.0(1.2)	1.3(1.2)	0.7(1.1)	0.6	
Felt uncomfortable	1.0(1.3)	0.7(1.0)	0.3(1.3)	0.2	
Family Conflict subscale (FC)	1.4(1.7)	1.0(1.2)	0.4(1.3)	0.3	0.004*
Argued with child	0.8(1.1)	0.7(1.0)	0.1(1.0)	0.1	
Caused conflict in the family	0.6(0.9)	0.3(0.6)	0.3(0.8)	0.4	
Financial Burden subscale (FB)	0.8(1.1)	0.6(0.8)	0.2(1.0)	0.2	0.095
Cause financial difficulties	0.8(1.1)	0.6(0.8)	0.2(1.0)	0.2	

Wilcoxon’s signed-rank test

*Significant at α = 0.05 level

^a^Calculated using Cohen’s d (= difference / SD)

In addition to the COHIP and FIS, global ratings of OHRQoL also known as single-item ratings, were used to assess the general oral health of the pediatric patients and their overall QoL. These questions were answered on a 6-point Likert scale from “Very bad” to “Very good”.

### Statistical analysis

Statistical analysis of the survey responses was performed using SPSS 21.0 (SPSS Inc., Chicago, IL, USA). The missing data was 4.39% of the total response. Before statistical analysis, the missing values of COHIP and FIS items were replaced by the variables’ means to obtain sum scores. Since there were no statistically significant differences in the number of decayed teeth and the results of the OHRQoL questionnaire between general anesthesia and intravenous deep sedation, we have performed statistical analysis with the combined results. First, the Cronbach’s alpha coefficient was used to measure internal consistency. As the Kolmogorov−Smirnov test indicated the COHIP-14 and FIS-12 scores did not follow a normal distribution, the Wilcoxon’s signed-rank test was utilized to compare OHRQoL pre- and post-treatment. Cohen’s d indicated the effect size and was calculated by dividing the average difference in OHRQoL scores between pre- and post-treatment by the standard deviation. An effect size of 0.2 < d ≤ 0.5 was considered small, 0.5 < d ≤ 0.8 was considered intermediate, and d > 0.8 was considered large. To assess convergent validity, the partial Spearman correlation was examined between the COHIP and global ratings and between FIS score and global ratings. The Wilcoxon’s rank-sum test was used to compare findings in patients with and without systemic disease and to compare individuals of different ages and genders. This test was also used to investigate effects of treatment variables, including number of decayed teeth, number of treated teeth and pulp treatment.

Finally, to understand the correlation between the utilized scales, a structural equation model was designed using IBM SPSS Amos 23.0.0 to build a Multi-indicator model. The hypotheses for the structural equation model were as follows. First, the subscales of COHIP and FIS could have different explanatory power on COHIP and FIS, and COHIP would have a significant explanatory power on FIS. The rationale for these hypotheses is that a chronic illness such as dental caries in children can affect the quality of life of the family, which is based on the family member’s recognition of chronic diseases in children [13]. Second, the magnitude of the explanatory power in the structure equation model would be different depending on the presence or absence of systemic disease. Accordingly, the individual SEMs for patients with and without systemic disease were constructed by confirmative factor analysis. This study included COHIP and FIS subscales as observed variables and COHIP-14 and FIS-12 per se as latent variables. To assess the fitness of the structural equation model, the chi-square *p*-value, Goodness of Fit Index (GFI), and Normed Fit Index (NFI) were calculated. In general, if the GFI and NFI values are above 0.9, the suggested model is appropriate and seems to have good explanatory power.

## Results

Analysis was carried out on data from 93 pediatric patients (46 males and 47 females) and their primary caregivers. Among caregiver participants, 91 (97.8%) were parents (81 mothers and ten fathers) and two (2.2%) were grandmothers. A mean age of the 93 pediatric patients was 5.0 ± 3.4 years. There were 43 patients without systemic diseases (21 male and 22 female) with a mean age of 4.0 ± 2.1 years, while the remaining 50 patients had systemic diseases (25 male and 25 female), and a mean age of 5.9 ± 3.9 years. Patients with systemic disease were significantly older than those without (*p* = 0.012).

The average dmft index and the average number of treated teeth due to dental caries were 10.8 ± 4.8 and 8.8 ± 4.4, respectively. There was no significant difference in dmft index or number of treated teeth between the groups with (10.6 ± 4.5, 8.7 ± 4.7) and without (11.0 ± 5.1, 9.0 ± 4.0) systemic diseases (*p* = 0.648, 0.640). Dental treatment included direct resin restoration, pulp treatment, prefabricated crown restoration and early extraction of carious teeth. The average number of teeth according to type of dental treatment was as follows: 5.6 ± 3.3 for direct resin restoration, 2.6 ± 2.8 for pulp treatment, 2.7 ± 2.9 for prefabricated crown restoration, and 0.5 ± 1.3 for early extraction. There was no statistically significant difference between the two groups according to type of treatment.

In the 16 patients excluded from the analysis, the mean age, the average dmft index and the average number of treated teeth were 5.8 ± 3.5, 11.8 ± 5.8 and 8.8 ± 4.4, respectively. These results were not statistically different from the results of the 93 patients included in the analysis (*p* = 0.426, 0.409 and 0.943, respectively).

Cronbach’s alpha coefficient, indicating internal consistency, for COHIP-14, OH, and FL were 0.737, 0.624, and 0.769, respectively. For FIS-12, PA, PE, and FC, the values were 0.866, 0.810, 0.770, and 0.532, respectively.

COHIP scores were higher and FIS scores were lower post-treatment than pre-treatment. Therefore, the absolute value of the difference between pre- and post-treatment scores was used. Each of the item, pre- and post-treatment scores, as well as the difference in scores for COHIP-14 and FIS-12 are outlined in Tables 1 and 2. COHIP-14 and its subscale OH and FL scores were significantly and clinically improved at post-treatment (all *p* <  0.001 and effect size = 1.0, 1.0, 0.5 respectively). Before dental treatment, the most frequent dental problem pointed out in OH was discoloration of the teeth (37.6%), while discomfort during mastication (33.3%) was indicated for FL.

FIS-12 and its subscale PA and PE scores were all significantly and clinically improved post-treatment (all *p* <  0.001 and effect size = 0.7, 0.6, 0.7). Before dental treatment, the most frequently reported concern in PA was “required more attention” (66.7%), while that in PE was “worried about less opportunity in future due to dental problems” (37.6%). In all the subcategories of FC and FB, more than half of the responders reported “never” (57.0, 60.2, 55.9%), or “almost never” (11.8, 20.4, 20.4%).

As shown in Table 3, partial Spearman correlations indicated statistically significant associations between the COHIP-14/FIS-12 scores and the global oral health status and overall QoL both before and after dental treatment. For COHIP-14 before treatment, r(s) = 0.438, *p* <  0.001 and r(s) = 0.241, *p* = 0.02, respectively, and for FIS-12 before treatment, r(s) = − 0.251, *p* = 0.015, r(s) = − 0.391, p <  0.001, respectively. For COHIP-14 after treatment, r(s) = 0.429, *p* <  0.001 and r(s) = 0.287, *p* = 0.005, respectively, and for FIS-12 after treatment, r(s) = − 0.396, p <  0.001, r(s) = − 0.372, p <  0.001, respectively. Correlations with the global oral health status were of moderate magnitude, and correlations with the overall QoL were of low magnitude.

**Table 3:** Partial Spearman correlations between COHIP-14 and FIS-12 scores and global oral health status and overall quality of life

	Global oral health status		Overall quality of life	
	*r(s)*	*p-value*	*r(s)*	*p-value*
COHIP-14 before dental treatment	0.438	< 0.001	0.241	0.02
FIS-12 before dental treatment	−0.251	0.015	−0.391	< 0.001
COHIP-14 after dental treatment	0.429	< 0.001	0.287	0.005
FIS-12 after dental treatment	−0.396	< 0.001	− 0.372	< 0.001

As shown in Table 4, the presence of systemic disease accompanied lower OHRQoL. Gender did not play a significant role in pre- and post-treatment scores of either the COHIP-14 or FIS-12 or in improvement level (*p* > 0.05). Age was not a significant factor for the FIS-12 score, but improvement in the COHIP-14 was significantly greater in patients aged 1−6 years than in those 7 years or older (8.8 ± 7.9, 3.6 ± 7.4, *p* = 0.012). And COHIP-14 and FIS-12 were not associated with number of decayed teeth, number of treated teeth, and pulp treatment before and after dental treatment, respectively (all *p* > 0.05). But more than five treated teeth and pulp treatment resulted in greater improvement in the COHIP-14 score (*p* = 0.016 and 0.024, respectively).

**Table 4:** COHIP and FIS scores according to gender, age, and medical condition of patients

		Gender		Age		Systemic Disease	
		Male	Female	< 7 years	≥ 7 years	Healthy^a^	Diseased^b^
		Mean(SD)					
COHIP-14	B	38.0(7.7)	37.0(7.1)	36.9(7.8)	39.6(8.3)	40.2(6.1)	34.9(8.6)*
	A	45.6(7.2)	44.7(8.3)	45.7(7.4)	43.3(9.0)	47.2(6.7)	43.3(8.2)*
	D	7.7(7.6)	7.7(8.6)	8.8(7.9)	3.6(7.4)*	7.0(6.9)	8.4(9.1)
FIS-12	B	16.0(9.3)	15.4(9.2)	15.2(9.4)	17.5(8.2)	11.5(6.9)	19.4(9.4)*
	A	10.2(8.6)	10.4(8.1)	9.4(8.0)	13.5(8.9)	6.5(5.5)	13.7(8.9)*
	D	5.7(6.8)	5.0(9.5)	5.8(8.1)	4.0(8.8)	5.0(5.0)	5.7(10.3)

Wilcoxon’s rank-sum test

B: Before treatment

A: After treatment

D in COHIP-14: Difference between B and A (A - B)

D in FIS-12: Difference between B and A (B - A)

Healthy^a^: Patients without systemic disease

Diseased^b^: Patients with systemic disease

*: Significantly different between groups (*p* < 0.05)

**Fig. 1:**
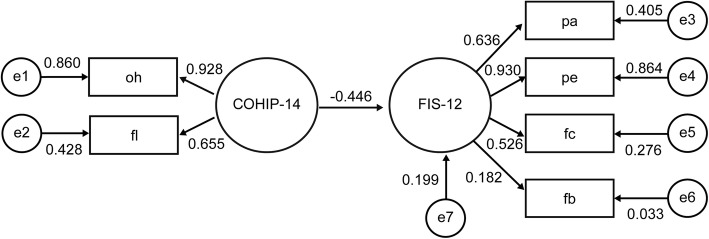
Structure Equation Model of COHIP and FIS in pediatric patients without systemic disease

**Fig. 2:**
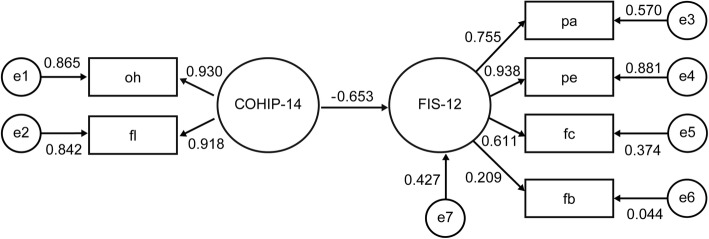
Structure Equation Model of COHIP and FIS in pediatric patients with systemic disease

Figures 1 and 2 shows the structure equation model flow-chart for pediatric patients without and with systemic disease respectively, as affected by COHIP and FIS variables. The coefficients estimated in this model represent the degree of explanatory power of the independent variable on the dependent variable, which indicates the degree to which the increment of one unit in independent variable changes the dependent variable including the error term. If the value is large, it has stronger explanatory power. The COHIP-14 score negatively affected the FIS-12 score, with explanatory power of 44.6 and 65.3% respectively. The reason for the negative direction is that higher COHIP and lower FIS scores indicate better OHRQoL. The magnitude of the explanatory power between COHIP and COHIP subscales and between FIS and FIS subscales was also greater in patients with systemic disease compared to patients without systemic disease. The chi-square test *p*-value, the GFI score and the NFI score were 0.807, 0.972, and 0.937 in the former model and were 0.060, 0.917, and 0.904 in the latter model, respectively. These results indicate exceptional fitness and explanatory power of the models.

## Discussion

This is the first study to examine the potential association between dental treatment and OHRQoL in pediatric patients in Korea, using a Korean version of the COHIP, which is the only questionnaire that has undergone reliability and validity testing in Korean pediatric patients [4]. OHRQoL is a subjective concept that relies strongly on patient’s awareness. Particularly in pediatric patients, teeth and facial development as well as psychological development vary markedly with age. The age of 6 years marks the beginning of abstract thinking and self-concept [23], and the understanding of even basic health concepts may be problematic in younger aged children, like the subjects of this study [24]. And pediatric patients with systemic disease often exhibit negative behavioral patterns during dental treatment due to the previous experiences in the medical hospital. They may also exhibit cognitive impairment, which makes it difficult to understand their cognitive processes and consequently results in unreliable measurement of QoL [7]. Therefore, studies on pediatric patients’ OHRQoL often rely on the awareness of their primary caregivers [25]. Previous studies have shown that there was greater agreement for observable oral conditions and lesser agreement for non-observable oral conditions between ratings of children’s OHRQoL made by parents and the children themselves [25]. In this context, the COHIP-14 and FIS-12 questionnaires used in this study were shortened from the original measurements for the primary caregiver to respond more appropriately. In order to supplement the modifications of the items, internal consistency and convergent validity were confirmed in this study.

The COHIP and FIS scores of pediatric patients were significantly and clinically improved after dental treatments under general anesthesia or intravenous deep sedation (Tables 1 and 2). These results were in agreement with those of previous studies that reported reduced OHRQoL due to a large number of dental caries [5, 6, 26] and assessed the OHRQoL of pediatric patients treated under general anesthesia [7–9, 16, 27–29]. These previous studies showed statistical and clinical improvements in all subscales including oral symptoms and function. And the effect size of FL was lower than that of OH, this is probably because the caregivers might have difficulties to recognize oral function objectively. This tendency is also observed in previous studies in which the caregivers responded to the questionnaire [7–9, 27, 29]. In contrast to these consistent results about dental caries on OHRQoL, the results of dental trauma and malocclusion, which are also common oral disorders in young pediatric patients, exhibited somewhat conflicting results [26, 30–32]. Overall, dental caries seems to have a greater impact on OHRQoL than dental trauma or malocclusion in young pediatric patients. This is likely because the OHRQoL questionnaires for young children are mostly completed by their caregivers, and dental caries in children are strongly influenced by the caregiver’s daily oral hygiene care, but the trauma and malocclusion are not directly related to the caregiver’s daily care.

Among the items in the COHIP, the most evident improvements were reported in the items of “discoloration of teeth” and “difficulty chewing firm food” before dental treatment, which are easy-to-notice changes for primary caregivers. This observation was slightly different from what was reported by Ahn et al. [4], where improvement in discoloration was reported at a low frequency, while improvements in food sticking in the teeth, crooked teeth, spaces between teeth, and difficulty in maintaining oral hygiene had high frequency. The age difference in the patient cohorts, as well as the targets of the investigation, patients versus primary caregivers, could account for the differences observed.

According to the study by Abanto et al. [5], dental caries exhibited a negative impact on the total FIS score and PA, PE and FB subscales. These are similar to our results except for the FB subscales. In our study, there was little change observed in the FB subscale, with the most frequent responses being “never” or “almost never” in items of the FB subscale. In cases where treatment is carried out under general anesthesia or intravenous deep sedation at Seoul National University Hospital, there is a possibility that the primary caregivers could either bear the financial burden for the treatment or received financial support from outside organizations. Among the items in the FIS, most evident improvements in this study were reported in the items of “required more attention” and “worried about less opportunity”. These results are also similar to those of Abanto et al..

The results of Table 4 indicate that OHRQoL is lower in patients with systemic disease before and after dental treatment. These findings were in accordance with previous reports that found that patients with systemic diseases, such as cerebral palsy [17, 33], autism [34, 35], cancer [10, 36], and craniofacial anomalies [37], suffered from a lower OHRQoL.

Gender was not an important factor in OHRQoL, in agreement with reports by Broder et al. in 2007 and de Paula et al. in 2015 [22, 38]. Greater improvement in the COHIP score in patients aged 6 years or younger may be related to the significantly higher average number of treated teeth compared to patients aged 7 years or older (9.9 vs. 4.9, *p* <  0.001). And this is consistent with the results that a large number of treated teeth and pulp treatments showed greater improvement in COHIP-14. A previous study reported that age is not an important factor, but that study did not consider the number of treated teeth when considering the effect of age [6].

The COHIP-14 appears to have a greater impact on the FIS-12 in patients with systemic disease (Figs. 1 and 2). Therefore, the diagnosis and understanding of oral health would exert a greater impact on the family’s OHRQoL for patients with systemic disease. In other words, dental treatment and improvement in oral health can result in an overall increase in the OHRQoL of families of patients with systemic disease.

General anesthesia or intravenous deep sedation was performed by a single anesthesiologist. However, dental treatment was performed by five professors in pediatric dentistry working at Seoul National University Dental Hospital. Therefore, the follow-up period varied among the dentists, resulting in inconsistent time-lapses between pre- and post-treatment surveys. To minimize the effect of the inconsistencies, only cases in which the duration between surveys was less than 6 months were included for analysis. In addition, we removed several items from original COHIP and FIS questionnaires to compensate the differences of patient age and respondents to the questionnaire. Despite of the validity and reliability tests conducted in this study, the COHIP-14 and FIS-12 were not fully validated. And the magnitude of correlations between the COHIP-14/FIS-12 and the overall QoL were low. This is probably because more than half of the patients had systemic disease, and systemic disease itself, apart from oral health status, could have a negative impact on the overall QoL. Therefore, further research to confirm the overall reliability and validity of COHIP-14 and FIS-12 are required. A difference in the mean age of patients with and without systemic disease and the fact that there was no equivalent cohort of children with similar systemic condition who were treated without general anesthesia or intravenous deep sedation may be other confounders.

## Conclusion

Dental treatment under either general anesthesia or intravenous deep sedation can improve the OHRQoL in Korean pediatric patients, which can be recognized by their primary caregivers. Systemic disease results in reduced OHRQoL, and the COHIP-14 appears to have a greater impact on the FIS-12 in patients with systemic disease than on patients without. In other words, the impact on the OHRQoL of the family is more pronounced in patients with systemic diseases, and thus treating dental caries in these patients will greatly improve the OHRQoL of the family members.

## Abbreviations

COHIP-14: Child oral health impact profile
FB: Financial burden subscale
FC: Family conflict subscale
FIS-12: Family impact Scale
FL: Functional limitation subscale
GFI: Goodness of fit index
NFI: Normed fit index
OH: Oral Health subscale
OHRQoL: Oral health-related quality of life
PA: Parental/family activity subscale
PE: Parental emotion subscale
